# Exercise cardiac MRI unmasks right ventricular dysfunction in acute hypoxia and chronic pulmonary arterial hypertension

**DOI:** 10.1152/ajpheart.00146.2018

**Published:** 2018-05-18

**Authors:** Shareen Jaijee, Marina Quinlan, Pawel Tokarczuk, Matthew Clemence, Luke S.G.E. Howard, J. Simon R. Gibbs, Declan P. O’Regan

**Affiliations:** ^1^MRC London Institute of Medical Sciences, Imperial College London, London, United Kingdom; ^2^Philips Healthcare, Philips Centre, Guildford, United Kingdom; ^3^Department of Cardiology, National Pulmonary Hypertension Service, Imperial College Healthcare NHS Trust, London, United Kingdom; ^4^National Heart and Lung Institute, Imperial College London, London, United Kingdom

**Keywords:** exercise testing, magnetic resonance imaging, pulmonary hypertension

## Abstract

Coupling of right ventricular (RV) contractility to afterload is maintained at rest in the early stages of pulmonary arterial hypertension (PAH), but exercise may unmask depleted contractile reserves. We assessed whether elevated afterload reduces RV contractile reserve despite compensated resting function using noninvasive exercise imaging. Fourteen patients with PAH (mean age: 39.1 yr, 10 women and 4 men) and 34 healthy control subjects (mean ageL 35.6 yr, 17 women and 17 men) completed real-time cardiac magnetic resonance imaging during submaximal exercise breathing room air. Control subjects were then also exercised during acute normobaric hypoxia (fraction of inspired O_2_: 12%). RV contractile reserve was assessed by the effect of exercise on ejection fraction. In control subjects, the increase in RV ejection fraction on exercise was less during hypoxia (*P* = 0.017), but the response of left ventricular ejection fraction to exercise did not change. Patients with PAH had an impaired RV reserve, with half demonstrating a fall in RV ejection fraction on exercise despite comparable resting function to controls (PAH: rest 53.6 ± 4.3% vs. exercise 51.4 ± 10.7%; controls: rest 57.1 ± 5.2% vs. exercise 69.6 ± 6.1%, *P* < 0.0001). In control subjects, the increase in stroke volume index on exercise was driven by reduced RV end-systolic volume, whereas patients with PAH did not augment the stroke volume index, with increases in both end-diastolic and end-systolic volumes. From baseline hemodynamic and exercise capacity variables, only the minute ventilation-to-CO_2_ output ratio was an independent predictor of RV functional reserve (*P* = 0.021). In conclusion, noninvasive cardiac imaging during exercise unmasks depleted RV contractile reserves in healthy adults under hypoxic conditions and patients with PAH under normoxic conditions despite preserved ejection fraction at rest.

**NEW & NOTEWORTHY** Right ventricular (RV) reserve was assessed using real-time cardiac magnetic resonance imaging in patients with pulmonary arterial hypertension and in healthy control subjects under normobaric hypoxia, which has been previously associated with acute pulmonary hypertension. Hypoxia caused a mild reduction in RV reserve, whereas chronic pulmonary arterial hypertension was associated with a marked reduction in RV reserve.

## INTRODUCTION

Pulmonary arterial hypertension (PAH) is a disease characterized by adverse remodeling of the peripheral pulmonary arteries leading to elevated pulmonary arterial pressure (PAP) and progressive right ventricular (RV) dysfunction (5, 17). In the early stages of PAH, the RV remains coupled to its afterload and maintains efficient energy transfer by a homeometric adaptive increase in wall thickness and contractility. With cumulative exposure to increasing afterload, heterometric adaptation occurs with progressive RV dilatation to preserve stroke volume (SV) until the more advanced stages of disease, when wall stress rises and ventriculoarterial uncoupling occurs with reduced cardiac output (26). It is known that RV function is a strong predictor of mortality, outperforming pulmonary vascular resistance (PVR) (18, 33, 35), but there is currently no method to determine which RV phenotype is destined to fail despite medical therapy. The change in systolic function on exercise or pharmacological stress is emerging as a more promising indicator of coupling efficiency (13), and the response of the pressure-loaded RV to exercise could be more relevant than resting hemodynamics for the followup and management of patients with PAH (11).

RV reserve can be assessed noninvasively during exercise using real-time imaging to provide a surrogate marker of stress-induced cardiopulmonary uncoupling (16, 21). Understanding the consequences of increased vascular load on RV contractile reserve may allow earlier risk stratification and evaluation of treatment response (38). In the present study, we used real-time exercise imaging to determine the effect of elevated afterload on RV contractile reserve during normobaric hypoxia (which has been associated with transient acute pulmonary hypertension) and in chronic PAH before the RV becomes dilated or impaired. We hypothesized that the RV has limited capacity to recruit contractile function when exposed to elevated afterload despite compensated function at rest.

## METHODS

### Study Participants

We prospectively enrolled patients undergoing routine treatment in the Pulmonary Hypertension Service at Imperial College Healthcare National Health Service Trust and normal subjects from August 2014 to February 2015. Ethical approval was given by the Health Research Authority, United Kingdom, and informed written consent was obtained from all participants. Patients were diagnosed with group 1 PAH according to joint European Society of Cardiology and European Respiratory Society guidelines and received standard treatment (8). Patients were excluded if they had a contraindication to cardiac magnetic resonance (CMR) imaging, exercise testing, were unstable, or had a resting RV ejection fraction (RVEF) of <45%.

### Study Design

Patients with PAH underwent CMR at rest and exercise while breathing room air, whereas normal subjects underwent an exercise protocol while breathing room air and then during normobaric hypoxia.

### Cardiopulmonary Exercise Test

The cardiopulmonary exercise test (CPET) comprised a ramp-protocol cycle test on an electronically braked bicycle ergometer (VIASprint 200, BD Worldwide). Normal subjects were encouraged to exercise until exhaustion, after which 3 min of rest and recovery data were recorded. Each participant breathed through a calibrated mass flow sensor with expired gas sampled on a breath-by-breath basis.

### Exercise Protocol

All participants performed a supine exercise protocol while lying in the bore of the magnet using an magnetic resonance-compatible cycle ergometer (Lode, Groningen, The Netherlands). CMR images were acquired with subjects breathing room air in the resting state and then at an exercise intensity threshold set at 40% of the watts achieved during upright CPET with a cadence of 60–70 revolutions/min. This protocol accounts for interindividual variation in exercise capacity as well as the difference in efficiency between upright (CPET) and supine (CMR) exercise (6), providing accurate assessment of cardiac output compared with the direct Fick method (21). In normal subjects, the protocol was repeated while subjects breathed 12% O_2_ (ensuring resting saturations had dropped and plateaued). The total duration of hypoxia was ~35 min.

#### CMR imaging.

CMR was performed on a 1.5-T Philips Achieva system (Best, The Netherlands), and a standard clinical protocol for structural, functional, and phase-contrast breath-hold imaging at rest was followed according to published international guidelines (20). Real-time imaging at both rest and exercise were then acquired using a previously validated ungated high-temporal resolution sequence during free breathing (Philips Healthcare Clinical Science, Surrey, UK) (21) using the following parameters: field of view, 305 × 305 mm; repetition time/echo time, 2.5/1.26 ms; flip angle, 50°; acquired voxel size, 2.73 × 2.73 × 10 mm; section thickness, 10 mm with no gap; reconstructed voxel size, 1.19 × 1.19 × 10 mm; number of sections, 14; slice scan order, base to apex; dynamic scan time, 66 s with 50 dynamics per slice; and temporal resolution, 74 ms. Free breathing, real-time main pulmonary artery and aortic phase-contrast images were acquired using the following parameters: field of view, 300 × 300; repetition time/echo time, 11/3.7 ms; flip angle, 20°; acquired voxel size, 3.0 × 3.0 × 10 mm; section thickness, 10 mm; reconstructed voxel size, 1.17 × 1.17 × 10 mm; dynamic scan time, 4.5 s with 100 dynamics; and temporal resolution, 44 ms.

#### Ventricular volumes and function analysis.

A standard clinical protocol for assessing biventricular function, volumes, mass, and flow was followed according to published international guidelines (20). Real-time imaging data were analyzed using a specially adapted proprietary software (cvi42, Circle Cardiovascular Imaging, Calgary, AB, Canada) and assessed during the expiratory phase of respiration. Volumes were indexed to body surface area calculated using the Mosteller formula. Indexed volumetric data were left ventricular (LV) and RV end-diastolic volumes (LVEDVi and RVEDVi, respectively), LV end-systolic volume and RV end-systolic volume (LVESVi and RVESVi, respectively), LV and RV stroke volumes (LVSVi and RVSVi, respectively), and LV ejection fraction (LVEF) and RVEF. Cardiac index (CI) was derived as follows: [LVSVi × heart rate (HR)]. RV contractile reserve was defined as RVEF with exercise − RVEF at rest (13). Real-time septal curvature measurements were assessed using previously described techniques (29).

### Reproducibility

Ten normal subjects returned for repeat exercise CMR imaging during normoxia within 3 mo of the initial scan for interstudy reproducibility. Inter- and intraobserver variability was assessed in 15 randomly selected data sets of both volume and flow at rest and exercise.

### Statistical Analysis

Data were analyzed using SPSS version 22 (IBM, Armonk, NY). Descriptive data for continuous variables are presented as means ± SD or as medians (interquartile ranges). Test-retest reliability was assessed using an intraclass correlation coefficient (ICC) with a two-way random model for absolute agreement. Comparisons between groups for continuous variables were performed using unpaired two-sample *t*-tests or the Mann-Whitney test and comparison between categorical variables with Fisher's exact test. Pearson product-moment correlation was used to assess the linear relationship between variables. The effect of O_2_ level and exercise on cardiac parameters was assessed with two-way repeated-measures ANOVA, and the effect of normoxic exercise in patients and normal subjects was compared with mixed ANOVA. The *F*-test remains a valid statistical procedure under non-normality in a variety of conditions (3). Interactions were first evaluated with simple main effects analysis. Predictors of RV reserve were assessed using stepwise multiple linear regression. Bonferroni correction was made for multiple comparisons. *P* values of <0.05 were considered significant.

## RESULTS

**Fig. 1.**
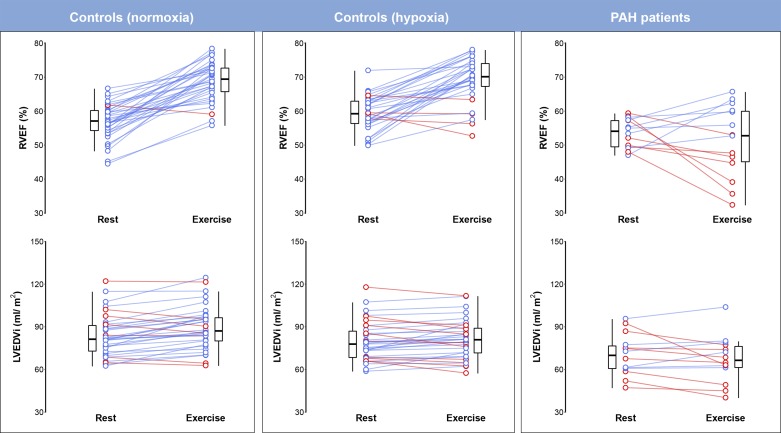
As assessed using real-time cardiac MRI, right ventricular reserve is mildly reduced by transient elevations of afterload in heathy subjects and markedly impaired in patients with chronic pulmonary arterial hypertension, which is associated with left ventricular underfilling. Exercise imaging offers a noninvasive means of identifying subjects in whom right ventricular contractile reserves are depleted. Tukey box and whisker plots with red lines show a decrease and blue lines show an increase in the parameter on exercise in individual subjects. LVEDVi, left ventricular end-diastolic volume index; PAH, pulmonary arterial hypertension; RVEF, right ventricular ejection fraction.

Thirty-eight normal subjects were investigated (age 35.5 ± 9.3 yr, 16 men and 12 women). Thirty-four subjects completed the protocol; one subject withdrew at the onset of the resting hypoxia and three subjects were excluded because they did not complete the hypoxic exercise protocol because of fatigue, and these normal subjects were excluded from the analysis. Fourteen patients with PAH were investigated (39.1 ± 9.4 yr, 4 men and 10 women) a mean of 3.9 ± 3.3 yr after the initial diagnosis. Thirteen patients had idiopathic PAH based on right heart catherization criteria, and one patient had hereditary PAH (heterozygous bone morphogenetic protein receptor type 2 mutation). CPET was performed 1.7 ± 2.2 mo before CMR, and the RHC data collected were at diagnosis. Baseline characteristics at diagnosis are shown in Table 1; treatments are shown in Table 2. Intra- and interobserver ICCs for resting RVEF were 0.71 and 0.85, respectively, and for exercise RVEF were 0.625 and 0.744. The interstudy ICC for exercise RVEF was 0.603. The effect of each exercise condition on RVEF and LVEDVi in patients and control subjects is shown in Fig. 1.

**Table 1.** Comparison of baseline characteristics of patients and normal subjects

	Patients With Pulmonary Arterial Hypertension	Control Subjects	*P* Value
Number of subjects/group	14	34	
Age, yr	39.1 ± 9.4	35.6 ± 9.3	0.13
Sex, men/women	4/10	17/17	0.21
Body surface area, m^2^	1.8 (0.2)	1.9 (0.3)	0.19
Body mass index, kg/m^2^	25.0 (8.4)	23.6 (4.0)	0.52
Maximal O_2_ consumption, ml·kg^−1^·min^−1^	1,412.0 (378.5)	2,798 (1055)	<0.0001
Max load CPET, W	115 (40.5)	241 (107)	<0.0001
Load CMR, W	43.5 (16.8)	92 (41)	<0.0001
Minute ventilation-to-CO_2_ output ratio, mL·kg^−1^·min^−1^	36.1 (8.4)		
World Health Organization functional class			
I	2		
II	8		
III	4		
6-min walking distance, m	446.9 (75.4)		
B-type natriuretic peptide, pg/ml	30.1 (19.6)		
Systolic PAP, mmHg	79.8 (27.0)		
Diastolic PAP, mmHg	31.7 (13.6)		
Mean PAP, mmHg	50.6 (17.6)		
Right atrial pressure, mmHg	7.6 (5.1)		
Pulmonary vascular resistance, Woods units	10.5 (6.3)		

Values are expressed as medians (interquartile ranges) and were compared with a Mann-Whitney test. Max load CPET, maximum watts achieved during cardiopulmonary exercise test; load CMR, level at which the subject was exercised during the cardiac magnetic resonance imaging; PAP, pulmonary arterial pressure.

**Table 2.** Summary of treatments in the patient group

Type of Therapy	Number of Patients	Specific Treatments
Anticoagulation	6	Warfarin
Monotherapy	2	Sildenafil Bosentan
Dual therapy	9	Sildenafil and ambrisentan Sildenafil and bosentan Sildenafil and macitentan Ambrisentan and tadalafil
Ca^2+^ channel blocker only	3	Diltiazem Nifedipine

### Biventricular Response to Normoxic and Hypoxic Exercise in Healthy Participants

**Fig. 2.**
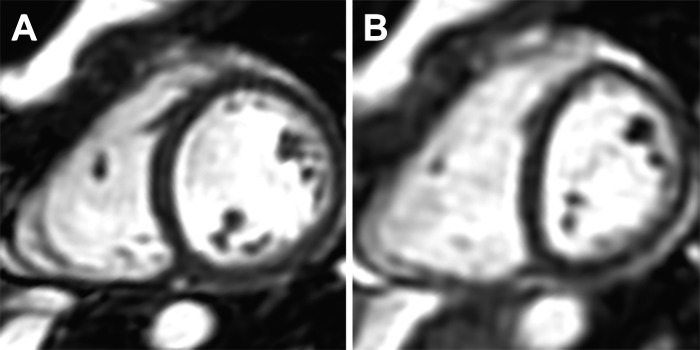
Cardiac magnetic resonance images of a healthy control subject in the left ventricular short-axis plane at end expiration during normoxia (*A*) and hypoxia (*B*), demonstrating septal flattening associated with hypoxia-induced pulmonary arterial hypertension.

Biventricular parameters during normoxia and hypoxia at rest and exercise are shown in Table 3. There was a significant effect of exercise on all cardiac parameters except RVEDVi (*P* between <0.0001 and 0.031). During normoxic exercise, normal subjects increased their CI by 2.5 times the resting value, at individualized workloads, with significant increases of HR, SV index (SVi), biventricular EF, and RVSVi/RVESVi. An increase in LVSVi was associated with increased end-diastolic volumes and decreased end-systolic volumes. In contrast, the increase in RVSVi was associated with decreased RVESVi but stable RVEDVi (all significant differences *P* < 0.0001). In normal subjects, breathing a fraction of inspired O_2_ of 12% reduced peripheral O_2_ saturation to 84 ± 4% at rest and 77 ± 5% during exercise with the development of concomitant interventricular septal flattening (*P* < 0.0001), which was present in 85% of subjects (Fig. 2). During resting hypoxia, HR increased by 21% and CI by 16% (*P* < 0.0001). Left atrial size at rest was significantly reduced by hypoxia (24.1 ± 5.4 to 21.0 ± 9.3 cm^2^, *P* < 0.05), but right atrial size was unchanged (24.8 ± 5.9 vs. 25.0 ± 5.4 cm^2^, *P* = 0.621). For most cardiac variables, the response to exercise depended on O_2_ level. There was no difference between resting SVi in normoxia and hypoxia (*P* = 0.76), but exercise SVi was significantly lower during hypoxia compared with normoxia (*P* < 0.001). The increase in RVEF on exercise was less during hypoxia (*P* = 0.017), but the response of LVEF to exercise did not depend on O_2_ level. Biventricular volumes decreased during resting hypoxia, and hypoxic exercise conditions accentuated the fall in RVEDVi and attenuated the rise of LVEDVi (*P* < 0.036 for all interactions).

**Table 3.** Cardiac parameters in healthy control subjects during rest and exercise in normoxia and hypoxia

	Rest		Exercise		*P* Values		
Variable	Normoxia	Hypoxia	Normoxia	Hypoxia	Exercise	Oxygen	Interaction
*Heart rate and flows*							
Heart rate, beats/min	61.6 ± 10.6	74.5 ± 12.2*	123.0 ± 10.5‡	142.8 ± 12.2*‡	<0.0001	<0.0001	0.004
Aortic stroke volume index, ml/m^2^	51.3 ± 10.9	50.4 ± 12.3	62.0 ± 10.3‡	58.3 ± 11.4†‡	<0.0001	0.009	0.048
Mean pulmonary artery stroke volume index, ml/m^2^	51.3 ± 9.9	51.6 ± 9.8	60.2 ± 9.3‡	56.2 ± 10.9†§	<0.0001	0.004	0.014
Cardiac index, l·min^−1^·m^−2^	3.1 ± 0.6	3.6 ± 0.7*	7.6 ± 1.5‡	8.3 ± 1.8*‡	<0.0001	<0.0001	0.328
*Left ventricular parameters*							
End-diastolic volume index, ml/m^2^	83.7 ± 14.6	79.9 ± 13.8*	89.6 ± 15.1‡	81.5 ± 13.6*	<0.0001	<0.0001	<0.0001
End-systolic volume index, ml/m^2^	30.2 ± 5.2	26.7 ± 5.6*	25.0 ± 6.7‡	20.6 ± 5.2*‡	<0.0001	<0.0001	0.375
Stroke volume index, ml/m^2^	53.5 ± 10.7	53.0 ± 10.1	64.6 ± 10.7‡	60.7 ± 10.3*‡	<0.0001	0.001	0.001
Ejection fraction, %	63.8 ± 3.6	66.6 ± 4.7†	72.2 ± 4.5‡	74.7 ± 4.3†‡	<0.0001	<0.0001	0.958
*Right ventricular parameters*							
End-diastolic volume index, ml/m^2^	90.0 ± 17.8	87.6 ± 16.7	88.3 ± 15.2	82.5 ± 15.4*	0.031	0.001	0.036
End-systolic volume index, ml/m^2^	38.6 ± 8.9	35.8 ± 9.4†	26.9 ± 8.7‡	25.6 ± 8.9‡	<0.0001	0.013	0.322
Stroke volume index, ml/m^2^	51.3 ± 11.1	51.7 ± 9.4	61.3 ± 10.0‡	56.7 ± 10.0*‡	<0.0001	0.013	<0.0001
Ejection fraction, %	57.1 ± 5.2	59.4 ± 5.1	69.9 ± 6.3‡	69.1 ± 6.6‡	<0.0001	0.305	0.017
Stroke volume index/end-systolic volume index	1.4 ± 0.3	1.5 ± 0.3†	2.5 ± 0.7‡	2.4 ± 0.7‡	<0.0001	0.585	0.086
*Left atrial parameters*							
Left atrial area, cm^2^	24.1 ± 5.4	21.0 ± 9.3†					
*Interventricular septal parameters*							
Interventricular septal curvature	0.9 ± 0.06	0.8 ± 0.1*					

*n* = 34 subjects/group. Two-way repeated-measures ANOVA with main effects of O_2_ level and exercise on each dependent variable was used. Simple main effects analysis between O_2_ levels was as follows:

* *P* < 0.0001 and

† *P* < 0.05. Simple main effects analysis between rest and exercise was as follows:

‡ *P* < 0.0001 and

§ *P* < 0.05.

### Biventricular Response to Exercise in Patients with PAH

Biventricular parameters in patients compared with control subjects are shown in Table 4. There were no significant differences in resting indexed RV volumes and function, HR, and CI between patients and normal subjects. During exercise, patients increased their CI by 1.8 times the resting value with a significant rise in HR. In contrast to control subjects, there was no overall response of RVEF to exercise, but the positive response of LVEF was not significantly different in patients. The lack of increase of RVSVi on exercise was associated with a marked increase in RVESVi compared with control subjects (*P* < 0.0001) despite a modest increase in RVEDVi (*P* < 0.001). RVSVi/RVESVi also showed no augmentation with exercise. Similarly, patients were unable to increase LVSVi despite a decrease in LVESVi on exercise, with no increase in end-diastolic volume.

**Table 4.** Cardiac parameters in patients with PAH during rest and exercise compared with healthy control subjects in normoxia

	Control Subjects		Patients With PAH		*P* Values		
Variable	Rest	Exercise	Rest	Exercise	Exercise	Between subjects	Interaction
*Heart rate and flows*							
Heart rate, beats/min	61.2 ± 10.8	122.4 ± 12.1‡	63.4 ± 11.5	114.1 ± 15.6‡	<0.0001	0.321	0.022
Aortic stroke volume index, ml/m^2^	51.4 ± 11.0	61.7 ± 10.4‡	45.7 ± 8.6	46.2 ± 12.1*	<0.0001	0.001	<0.0001
Mean pulmonary artery stroke volume index, ml/m^2^	51.4 ± 9.9	60.0 ± 9.3‡	40.0 ± 8.0	40.0 ± 13.3*	0.001	<0.0001	<0.0001
Cardiac index, l·min^−1^·m^−2^	3.1 ± 0.6	7.6 ± 1.5‡	2.9 ± 0.6	5.2 ± 1.2*‡	<0.0001	<0.0001	<0.0001
*Left ventricular parameters*							
End-diastolic volume index, ml/m^2^	84.3 ± 14.7	90.1 ± 15.5‡	70.3 ± 14.6†	67.2 ± 16.3*	0.264	<0.0001	0.001
End-systolic volume index, ml/m^2^	30.4 ± 5.3	25.5 ± 7.0‡	25.7 ± 9.3	18.4 ± 8.8‡	<0.0001	0.005	0.169
Stroke volume index, ml/m^2^	53.8 ± 10.7	64.6 ± 10.7‡	44.6 ± 6.7†	48.9 ± 9.1*§	<0.0001	<0.0001	<0.0001
Ejection fraction, %	63.7 ± 3.6	71.9 ± 4.5‡	64.3 ± 6.2	73.7 ± 6.7‡	<0.0001	0.363	0.405
*Right ventricular parameters*							
End-diastolic volume index, ml/m^2^	89.6 ± 2.7	85.8 ± 4.4	88.1 ± 2.5	93.2 ± 4.0*§	0.024	0.890	0.001
End-systolic volume index, ml/m^2^	38.4 ± 8.5	27.1 ± 8.4‡	40.1 ± 9.5	45.8 ± 15.9†	0.056	0.001	<0.0001
Stroke volume index, ml/m^2^	51.2 ± 1.6	60.9 ± 1.6‡	45.3 ± 2.6	47.4 ± 2.6*	<0.0001	0.002	<0.0001
Ejection fraction, %	57.1 ± 5.2	69.6 ± 6.1‡	53.6 ± 4.3	51.4 ± 10.7*	<0.0001	<0.0001	<0.0001
Stroke volume index/end-systolic volume index	1.4 ± 0.3	2.4 ± 0.7‡	1.2 ± 0.2†	1.1 ± 0.5*	<0.0001	<0.0001	<0.0001
End-systolic volume/stroke volume	0.8 ± 0.2	0.4 ± 0.1‡	0.9 ± 0.1	1.0 ± 0.5§	0.071	<0.0001	<0.0001

*n* = 14 patients with PAH and 38 healthy control subjects. PAH, pulmonary arterial hypertension. Mixed ANOVA for comparing the exercise-dependent change in each variable between groups was used. Simple main effects analysis compared with control was as follows:

* *P* < 0.001 and

† *P* < 0.05. Simple main effects analysis from rest to exercise was as follows:

‡ *P* < 0.001 and

§ *P* < 0.05.

### Correlations and Predictors of RV Reserve in Patients with PAH

While all but one of the normal subjects increased their RVEF on normoxic exercise, only 50% of patients with PAH could achieve this despite there being no difference in mean resting RVEF between groups. There was no significant difference in the following parameters between those who increased or decreased RVEF on exercise: years since diagnosis, 2.0 (6) versus 3.0 (5) yr, *P* = 1.0; PVR, 6.3 (2.9) versus 17.0 (12.1) Woods units, *P* = 0.612; mean PAP at diagnosis, 42 (15) versus 69.0 (23) mmHg, *P* = 0.612; most recent 6-min walk distance, 456 (120) versus 432 (82) m; maximum O_2_ uptake, 1,600 (293) versus 1260 (205) ml·kg^−1^·min^−1^, *P* = 0.52; minute ventilation-to CO_2_ output ratio, 33 (12.4) versus 36.7 (5.6) ml·kg^−1^·min^−1^, *P* = 1.0; or B-type natriuretic peptide, 18.1 (14) versus 35.5 (40.9) pg/ml, *P* = 1.0. Peak O_2_ uptake correlated moderately with RV reserve (exercise RVEF − resting RVEF, *r* = 0.517, *P* < 0.0001), LVEDVi (*r* = 0.513, *P* < 0.0001), and exercise RVEF (*r *= 0.548, *P* < 0.0001) and more strongly with RVSVi and LVSVi (*r *= 0.722, *P* < 0.0001; *r* = 0.770, *P* < 0.0001). Of baseline RHC, CPET, and 6-min walk distance variables only, the minute ventilation-to CO_2_ output ratio was an independent predictor of RV reserve (*P* = 0.021). The RVESV-to-RVSV ratio has been proposed as a noninvasive estimate of ventricular-vascular coupling (30). We found that this ratio was higher on exercise in those with impaired contractile reserve compared with those with preserved contractile reserve (1.4 ± 0.4 vs. 0.6  ± 0.1, *P* = 0.001), suggesting an uncoupling response to exercise.

## DISCUSSION

We investigated biventricular contractile reserve using real-time CMR during acute normobaric hypoxia in healthy adults and in patients with compensated PAH (RVEF > 45%). Noninvasive cardiac imaging during exercise unmasks reduced RV contractile reserve in healthy adults under hypoxic conditions and a marked depletion of reserve in patients with PAH under normoxic conditions despite preserved resting systolic function. Exercise imaging of the pressure-loaded right heart during exercise offers a means to stratify patients with normal resting function noninvasively.

A moderate increase in pulmonary vascular tone over the first 5 min of normobaric hypoxia has been observed in heathy adults at rest using Doppler echocardiography (34), and a similar response has been observed in animal models using invasive measurements (39). As the pulmonary vascular pressure-flow relationship is steep, there is a substantial afterload on the RV during exercise (26). Resting hypoxia induced a significant decrease in peripheral O_2_ saturation in our normal subjects, which further decreased during hypoxic exercise. We observed that the increase of RVEF on exercise in normal subjects was attenuated by acute hypoxia and was associated with a fall in SVi despite sympathetic activation and homeometric adaptation. In contrast, the positive response of LVEF to exercise remained the same during hypoxic and normoxic exercise. Our data show that hypoxia causes mildly impaired contractile reserve selectively affecting the RV, although the RVSVi-to-RVESVi ratio suggested that coupling was maintained (37). Sildenafil may reverse the negative impact of hypoxia on RV contractility during exercise in some subjects (19), and taken with our findings, it is plausible that the RV limits exercise capacity during hypoxic vasoconstriction (26).

During exercise, an increase in mean PAP is partially offset by a decline of PVR in healthy adults, but in patients with PAH, PVR is fixed, placing even greater contractile demands on the RV (27). Furthermore, in the context of PAH, contractile reserve is dependent on multiple factors, such as ventricular contractility, myocardial fibrosis, and response to neurohormonal activation (13). Previous imaging studies in patients with PAH with dilated and impaired RVs at rest have shown impaired cardiac performance during exercise (14, 23), but our data show that diminished contractile reserves are also a characteristic of patients with preserved resting RV systolic function and volumes, with up to half of patients exhibiting a fall in RVEF on submaximal exercise. In normal subjects, the increase in SVi on normoxic exercise was most likely determined predominantly by enhanced contractility (↓RVESVi ≫ ↓RVEDVi). In contrast, patients with PAH were not able to augment SVi, with RVEDVi and RVESVi both shifting by similar amounts to higher operating volumes suggestive of uncoupling of the RV and pulmonary arteries (15). While there are several mechanisms by which contractile reserve might be impaired in PAH, our data from hypoxic exercise indicates that even the healthy RV has limited capacity to recruit contractile function in the face of rising afterload. There is a strong relationship between ventricular-arterial coupling and RV contractile reserve in animal models of chronic pressure overload (12), supporting the potential role of stress testing as a surrogate marker for exercise-induced uncoupling in PAH (12, 16, 32). Exercise-induced changes in RVEDVi and pressure-mediated septal curvature interfere with LV diastolic filling, but our data also suggest that an impaired response to exercise may contribute to reduced LV preload and consequent underfilling.

Clinical deterioration in PAH is preceded by alterations in resting RV volumes (36), and progressive loss of function is associated with a poor outcome, irrespective of any changes in PVR (1, 18, 33, 35). Conventional assessment of baseline function is often a weak predictor of the transition from a compensated state to right heart failure, and hence detecting RV dysfunction using exercise MRI before it manifests as resting RV dilatation and dysfunction could unmask ventricular-vascular uncoupling (9, 25, 31, 40). Using exercise imaging to risk-stratify patients by RV contractile reserve and to determine response to therapy is worthy of further investigation and would offer a noninvasive alternative to the hemodynamic stress tests, which are known to be strong predictors of prognosis in left heart failure, PAH, and chronic thromboembolic pulmonary hypertension (4, 7, 13). Previous studies have also implicated poor RV performance and higher exercise pulmonary vascular tone in the pathogenesis of ventilatory inefficiency (22), and we have shown that the minute ventilation-to CO_2_ output ratio is independently associated with RV contractile reserve in PAH. The mechanism by which dynamic changes in PVR during exercise mediate inefficient ventilation is debated, but the minute ventilation-to CO_2_ output ratio has been shown to be the single best indicator of increased risk of adverse events in heart failure (28) and is predictive of outcome at baseline in PAH (10). Impaired RV function may contribute to ventilatory inefficiency and exercise-related dyspnea even in well-compensated patients with PAH.

### Limitations

Exercise CMR using ungated continuous imaging is considered the “gold standard” for ventricular volume measurement during high-intensity exercise and shows close agreement with invasive assessment, but its accuracy depends on adequate temporal and spatial resolution to avoid underestimating SV at high HRs (21, 24). The best metric of contractile reserve is still an area of investigation, and although inotrope-induced change in ventricular elastance is the reference standard, in clinical practice contractile reserve is usually defined by a change in EF or SV during exercise or with dobutamine (13). Acute hypoxia leads to rapid pulmonary vasoconstriction and has been used in numerous studies of normal subjects, at rest and exercise, to investigate how the respiratory and cardiovascular systems respond to acute rises in pulmonary pressures (2, 26, 39). However, acute hypoxia not only directly affects the vascular tone of the pulmonary and systemic vessels but also causes sympathetic nervous activation leading to enhanced HR, cardiac output, and myocardial contraction (27). In hypoxia, septal curvature measurements indicated a significant rise in the transeptal pressure gradient, but we did not measure pulmonary hemodynamics during CMR.

### Conclusions

Noninvasive cardiac imaging during submaximal exercise demonstrates that RV contractile reserve decreases during hypoxic vasoconstriction in healthy control subjects and that patients with chronic PAH show marked depletion of contractile reserves despite preserved EF at rest. Exercise imaging has the potential to unmask dysfunction in the RV before resting adaptations to PAH are evident.

## GRANTS

The work was supported by British Heart Foundation Project Grant PG/13/44/30321, the National Institute for Health Research Biomedical Research Centre based at Imperial College London Healthcare NHS Trust and Imperial College London, and the Medical Research Council, United Kingdom.

## DISCLOSURES

No conflicts of interest, financial or otherwise, are declared by the authors.

## AUTHOR CONTRIBUTIONS

S.J., M.C., L.S.H., J.S.R.G., and D.O. conceived and designed research; S.J., M.Q., and P.T. performed experiments; S.J. and P.T. analyzed data; S.J. interpreted results of experiments; S.J. and D.O. prepared figures; S.J. drafted manuscript; S.J., L.S.H., J.S.R.G., and D.O. edited and revised manuscript; S.J., M.Q., P.T., M.C., L.S.H., J.S.R.G., and D.O. approved final version of manuscript.
